# Examining acute environmental effects on affective state, expectancy, and intention in sport climbing

**DOI:** 10.3389/fpsyg.2023.1258121

**Published:** 2023-12-14

**Authors:** Benedikt Hösl, Martin Niedermeier, Martin Kopp

**Affiliations:** Department of Sport Science, University of Innsbruck, Innsbruck, Austria

**Keywords:** physical activity, sport climbing, green exercise, affective state, expectancy, intention

## Abstract

**Introduction:**

Psychological research has shown that, among other variables, affective state, expectancy, and behavioral intention influence whether or not a (physically active) behavior is performed. Environmental effects during physical activity on affective state have been well studied; however, research regarding environmental effects on expectancy or intention is limited. Sport climbing is a form of physical activity that is performed both indoors and outdoors and is therefore considered as suitable to study environmental effects. Therefore, the aim of the present study was to investigate environmental effects during sport climbing on affective state, expectancy, and intention. The nature of the relationship between some of these parameters should also be explored.

**Methods:**

Using a within-subjects design, 48 participants were to climb both once indoors in a climbing gym and once outdoors at a crag. The design included questionnaire-based surveys at multiple time points. Affective state, expectancy, and behavioral intention were measured at different test time points.

**Results:**

Two-factor repeated-measures ANOVAs revealed significant main effects of the factors environment (indoor – outdoor) and time (T1 – T2 – T3) for affective state, indicating more positive affective state during outdoor climbing. No environment*time interactions were found. Furthermore, significantly higher intention (*d* = 0.32; *p* = 0.032) was measured after the outdoor condition (*M* = 32.5, SD = 4.1) compared to the indoor condition (*M* = 31.2, SD = 4.7). Multiple linear regressions revealed that expectancies (measured before and after the session) significantly predicted post-climbing intention only indoors.

**Conclusion:**

The results at least partly suggest environmental effects on behaviorally relevant variables during climbing. Outdoor climbing might provide more favorable characteristics for physically active behavior compared to indoor climbing. Health psychologists or public health professionals who focus on increasing physical activity for their clients could recommend outdoor climbing over indoor climbing.

## 1 Introduction

Although there is much evidence that physical activity has beneficial effects on physical and mental health (Goodwin, 2003; Lear et al., 2017), the majority of the population is not sufficiently physically active (Finger et al., 2017; World Health Organization, 2021). For this reason, it is advisable to take a closer look at the determinants of physical activity or focus on the circumstances in which physically active behavior can be increased. According to psychological research, variables that influence whether or not a (physically active) behavior is performed include affective state, expectancy, and behavioral intention (Gellert et al., 2012; Rhodes and Kates, 2015; Ungar et al., 2021; Bichler et al., 2022). Affective state can be characterized in terms of the two basic dimensions affective valence and affective arousal or activation (Russell, 1980). According to this, one feels rather pleasant or unpleasant and at the same time more or less activated (Brand and Schweizer, 2019). In this regard, positive affective state during or after physical activity in particular can positively influence the likelihood of future active behavior (Williams et al., 2008; Rhodes and Kates, 2015). Ekkekakis et al. (2011) assume that especially states of positive valence and high activation increase this likelihood. That physical activity can have positive effects on the affective state has been demonstrated several times, as a consequence of both multiple bouts (Reed and Buck, 2009) and single bouts of physical activity (Niedermeier et al., 2021). Sport climbing describes climbing along a given climbing route, whereby the climber is secured using appropriate climbing equipment. Compared to alpine climbing routes, sport climbing routes are generally shorter and better secured. The focus is more on the sporting aspect and less on climbing a specific wall, such as in alpine climbing. A further distinction must be made between bouldering, which describes a rope-free type of climbing in which one generally does not climb above jump height (Austrian Alpine Association, 2002). Sport climbing requires a combination of strength and endurance, with general aerobic endurance being trained to a lesser extent (Schweinheim and Schmied, 2011). In addition, climbing demands a high degree of agility, concentration, coordination, balance or orientation skills and is characterized by its psychological qualities (Schweinheim and Schmied, 2011; Leichtfried, 2015). Even though there is little literature on this subject for climbing, more positive affect or affective valence was also found after single bouts of climbing, predominantly in clinical settings (Frühauf et al., 2020; Bichler et al., 2022). In addition to physical activity, affective changes due to the environments should be considered. Research suggests that green exercise, which is physical activity performed in nature or natural environments, may enhance these positive effects on the affective state (Thompson Coon et al., 2011; Niedermeier et al., 2017).

Other variables that contribute to whether a certain behavior will occur relate to a person’s expectancy as well as intention; these play an important role in some theories of health behaviors, which include physical activity (Schwarzer, 2004). According to the Health Action Process Approach (HAPA) by Schwarzer (1992) in addition to self-efficacy expectancy, outcome expectancies in particular influence the formation of behavioral intention (motivational phase). Self-efficacy refers to the belief that one is able to successfully handle challenging situations based on one’s own competence (Bandura, 1997). Outcome expectancies are defined as expectations about consequences that may result from a certain behavior (Fuchs, 1994) and can be characterized using the dimensions of temporal proximity and area of consequences (Schwarzer and Luszczynska, 2016). Temporal proximity describes when the respective consequences of the behavior are expected, i.e., rather short-term (proximal) or rather long-term (distal). Area of consequences means whether physical, self-evaluative (affective) or social consequences are anticipated. According to the HAPA, intention affects behavior indirectly through planning processes; the target behavior itself is in turn influenced by other variables, such as resources, barriers, or again self-efficacy. This means that an intention does not necessarily lead to behavior. The fact that self-efficacy is useful for predicting physical activity or the intention to do so has already been described (Barg et al., 2012; Zhang et al., 2019). Furthermore, outcome expectancies or intention were also found to directly predict physical activity (Gellert et al., 2012; Ungar et al., 2021). In this regard, some findings from research suggest that proximal outcome expectancies may be more important in terms of behavioral motivation than distal outcome expectancies. Kraft et al. (2005) found that affective expectancies played a more important role than instrumental ones in predicting physical activity intention. Gellert et al. (2012) concluded that particularly expectations related to affective states during or immediately after physical activity predict physical activity at a later time, and less such expectancies that focus on health-related outcomes. Klusmann et al. (2016) found, that in the context of a long-term increase in physical activity, again proximal outcome expectancies (these focus more on short-term, affective consequences of a behavior) seem to play an important role. The authors identified the fulfillment of outcome expectancies to be a strong predictor of whether a certain (health-beneficial) behavior is successfully implemented or not. Thus, when examining immediate effects related to outcome expectancies in the context of physical activity, it seems important to focus more on proximal, affective expectancies and less on distal expectancies.

While there are several indications on positive effects of natural environments on affective state (Niedermeier et al., 2017; Lahart et al., 2019), information on the influence of environmental factors on expectancy or intention is not consistent. In this regard, some authors again suggest that green exercise may be associated with stronger intention to repeat exercise in the future. Focht (2009) reported that even a 10-minute walk can produce improvements in affective state both indoors and outdoors. On the other hand, participants reported in connection with outdoor walking more pleasant affective states, greater enjoyment, and stronger intention for future walking compared to an indoor walk on a treadmill. Also, Thompson Coon et al. (2011) concluded that green exercise is probably associated with stronger behavioral intention. However, some authors assume that green exercise does not have such a reinforcing effect on intention (Rogerson et al., 2016; Lahart et al., 2019), which is why there is no consensus on this issue.

To the best of the authors’ knowledge, no study has examined indoor and outdoor climbing in regard to the constructs affective state, expectancy, and intention. Therefore, the purpose of this study was to compare indoor and outdoor climbing in terms of affective state, expectancy, and intention. The nature of the relationship between some of these parameters should also be explored from which further recommendations and strategies related to public health could be derived. To this end, the following research questions were stated: First, does a sport climbing session have positive effects on the affective state? Based on existing literature, we hypothesized that an outdoor session would have greater positive effects on affective state than an indoor session. Second, we wanted to examine, whether there is an environmental effect on intention. Hereby we hypothesized that participants would have a stronger behavioral intention after the outdoor climbing session than after the indoor session. Third, we wanted to know if there is a positive relationship between expectancy (proximal outcome expectancies, self-efficacy) and intention in sport climbing.

## 2 Materials and methods

### 2.1 Design and procedure

**Figure 1:**
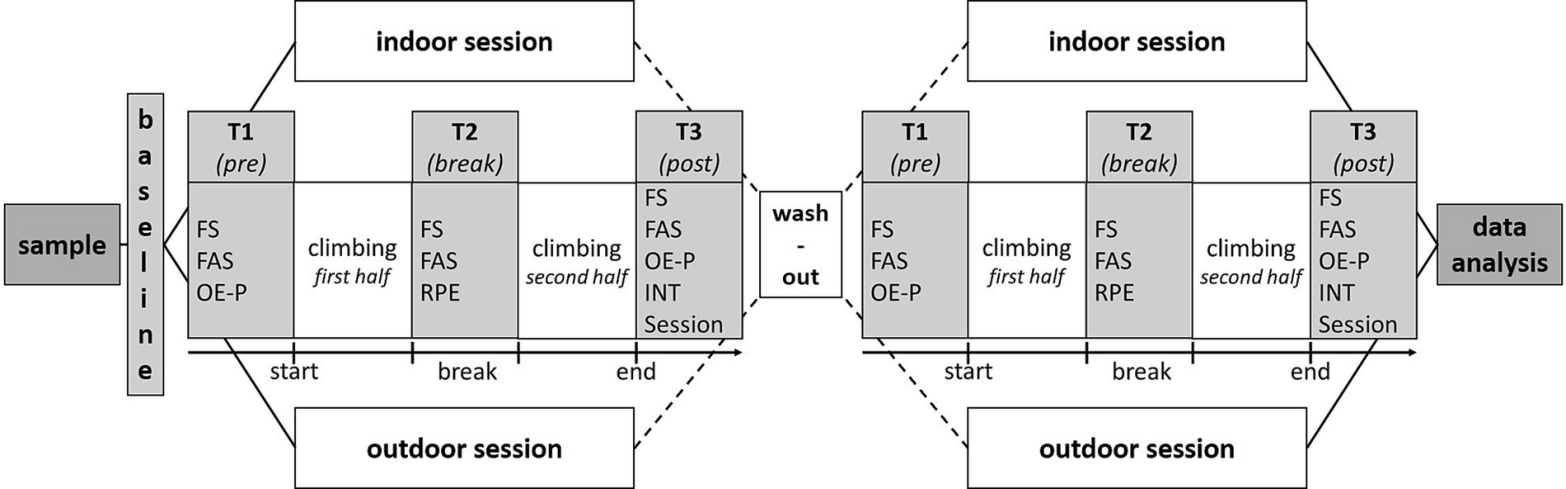
Research design including measurement times during the climbing sessions. FS, Feeling Scale; FAS, Felt Arousal Scale; OE-P, proximal outcome expectancies; RPE, Ratings of Perceived Exertion; INT, intention; SESSION, information about the completed session.

A within-subjects crossover design was chosen for the study. In this context, participants were asked to complete both an indoor and an outdoor climbing session at the crag independently (without an instructor). All data were collected by means of online questionnaires, which the participants had to fill out at different test times. The exact procedure was as follows (Figure 1): Participants first completed a baseline measurement and then started with one of the two conditions (indoor or outdoor climbing session). After completing the first session, the second, complementary session should be carried out within a period of 2 to 7 days (washout phase). For each condition, participants were instructed to climb a total of four routes. After the first two routes had been climbed, participants were to take a 30-minute break (long break) before climbing the remaining two routes. Immediately before each climbing session (T1), at the beginning of the long break (T2), and immediately after each session (T3), subjects were to complete a questionnaire.

In order to reduce dropout, the participants could choose which climbing condition to start with and when to do it. Thereby a natural random order was sought. However, the period of data collection had to be considered (September 2020 to October 2021).

In the baseline measurement, climbing-specific data were collected in addition to sociodemographic data, as well as participants’ self-efficacy expectancy.

Figure 1 also shows the data collection during the climbing sessions. In questionnaire T1, the dimensional affective state (Feeling Scale & Felt Arousal Scale) was assessed as well as proximal outcome expectancies for the respective upcoming climbing session. At T2, in addition to affective state, perceived intensity (Ratings of Perceived Exertion) was measured. In questionnaire T3, affective state, proximal outcome expectancies for the next (future) climbing session, behavioral intention, and information on the completed climbing session were assessed.

The study design including all test instruments was approved in advance by the Review Board of the Institute of Sport Science of the University of Innsbruck in accordance with the Declaration of Helsinki (#41/2020, 01.09.2020). Informed consent was required for participation in the study.

### 2.2 Sample

The required sample size was calculated *a priori* using G*Power (Faul et al., 2007). Based on previous research (Niedermeier et al., 2017), the expected effect size was set at *d* = 0.54. Accordingly, a sample size of *n* = 34 was targeted for the calculation of a 2 (environment: outdoor - indoor) × 3 (time: T1 - T2 - T3) repeated measures analysis of variance (ANOVA), with a power of 0.80 at a significance level of *α* = 0.05 and a correlation among repeated measures of *r* = 0.3. If a dropout rate of 30% is included, the minimum sample size of subjects to be recruited was *n* = 45.

The acquisition of participants took place partly online (mail, social networks), but also by personal recruitment in different climbing areas around Innsbruck and Tyrol, Austria. Participation was on a voluntary basis and could be canceled at any time. All data were collected pseudonymized.

The following inclusion criteria were communicated at the baseline questionnaire: (a) participation in sport climbing with rope, both indoors and outdoors on rock as a basic requirement, (b) appropriate belaying and climbing techniques, (c) age 18 years and above, and (d) German language skills, as the questionnaires were formulated in German.

Finally, 48 persons provided complete data sets. The average age of the 48 participants was 26.9 ± 3.7 years. The majority of participants indicated a male gender (62.5%) and a German nationality (60.4%). Further sociodemographic characteristics of the sample can be found in Table 1.

**Table 1:** Sociodemographic characteristics of the sample.

Variable	%	*n*
Gender		
Female	37.5	18
Male	62.5	30
Education		
Compulsory school	2.1	1
Vocational school (apprenticeship)	6.3	3
Further school education without Matura/A-levels	6.3	3
Further school education with Matura/A-levels^a^	20.8	10
University, technical college	64.6	31
Nationality		
Austria	39.6	19
Germany	60.4	29

^a^ Those schools provide a degree which allow students to attend a University.

On average, the participants reported 7.2 ± 5.8 years of indoor and 6.1 ± 5.9 years of outdoor climbing experience. The weekly climbing time was 4.9 ± 3.5 h. The redpoint climbing level of the sample ranged from grades 4a - 8a (median: 6b) on the French scale of difficulty, and the onsight level ranged from 4a - 7c (median: 6a).

### 2.3 Measurements

#### 2.3.1 Affective state

Affective state was measured dimensionally. Based on the Circumplex Model of Affect (Russell, 1980), the two dimensions valence and perceived activation were used for operationalization. Affective valence was measured using a German version of the Feeling Scale (Hardy and Rejeski, 1989). The instrument has only one item and thus offers the advantage of a short processing time. A bipolar, 11-point Likert scale is used to indicate how good or bad one feels at the moment. The response format ranges from +5 “very good” to −5 “very bad,” with the response option 0 forming the neutral anchor (Maibach et al., 2020). Regarding convergent validity between the Feeling Scale (German version) and the dimension Valence of the Self-Assessment Manikin (Bradley and Lang, 1994), correlation coefficients of *r* = 0.72–0.73 were found (Maibach et al., 2020).

Perceived activation was assessed using a German version of the Felt Arousal Scale (Svebak and Murgatroyd, 1985). The instrument also includes only one item, which can be answered on a six-point bipolar scale. Response options range from 1 “low arousal” to 6 “high arousal” (Maibach et al., 2020). Correlation coefficients of *r* = 0.50–0.62 were found (Maibach et al., 2020) between the Felt Arousal Scale (German version) and the Activation dimension of the Self-Assessment Manikin (Bradley and Lang, 1994).

#### 2.3.2 Outcome expectancies

Proximal outcome expectancies focus on more short-term consequences of a behavior. These were assessed using a total of eight items. For the present study, four already existing items were used, which can be found in Klusmann et al. (2016). These items primarily target affective outcomes of a behavior. Analogously, four further items were generated. Different instructions were used depending on whether proximal outcome expectancies were assessed before or after the climbing session. The instruction regarding proximal outcome expectancies collected before the climbing session referred to the respective upcoming session (“Please rate the following expectancies for today’s sport climbing session”). The instruction regarding proximal outcome expectancies assessed after the climbing session referred to the next session that the subjects would perform (“Please rate the following expectancies for your next sport climbing session”). An example item is: “When I do the next climbing session, I feel more balanced.” A five-point scale was used to answer the items, ranging from 1 “strongly disagree” to 5 “strongly agree.” To calculate the total score, the scores for each of the eight items were added up, resulting in a minimum score of 8 (low proximal outcome expectancies) and a maximum score of 40 (high proximal outcome expectancies). Internal consistency of the scale was calculated in the course of this study for both the indoor and outdoor condition. Regarding the outcome expectancies obtained before the climbing session, internal consistency was Cronbach’s *α* = 0.86 for the indoor condition and Cronbach’s *α* = 0.79 for the outdoor condition. For the outcome expectancies measured after the climbing session, Cronbach’s *α* was 0.77 for both the indoor and outdoor condition.

#### 2.3.3 Intention

To determine behavioral intention, a total of 10 items were created based on Schwarzer (2008) and Schwarzer et al. (2007). The items were formulated specifically for sport climbing. An example item is: “I intend to go climbing in my free time on a regular basis.” To answer the items, a four-point scale was used, ranging from 1 “strongly disagree” to 4 “strongly agree.” To calculate the scale score, the values of the 10 items are summed, resulting in a minimum score of 10 (low intention for future sport climbing) and a maximum score of 40 (high intention for future sport climbing). The internal consistency for the indoor condition was *α* = 0.84 and outdoors *α* = 0.76.

#### 2.3.4 Self-efficacy expectancy

To measure self-efficacy in the context of climbing, a questionnaire called Selbstwirksamkeit zur sportlichen Aktivität by Fuchs and Schwarzer (1994) was used, which is particularly suitable for studies in the field of health and sport psychology. The questionnaire aims at the beliefs of a person to be able to stick to a sporting activity even under adverse circumstances and includes 12 items that are to be assessed by means of a seven-point scale (from 1 “not sure at all” to 7 “quite sure”). The instruction was adapted for sport climbing. An example item is: “I am sure that I will still be able to perform a planned sports activity (sport climbing) even if I cannot find anyone to do sports with me.” The total score for each participant is the arithmetic mean across all 12 items, resulting in a minimum score of 1 (low self-efficacy) and a maximum score of 7 (high self-efficacy). The internal consistency of the scale is *α* = 0.89. Further psychometric parameters can be found in Fuchs and Schwarzer (1994). In the context of this study, an internal consistency of *α* = 0.71 was obtained.

#### 2.3.5 Perceived exertion

Since the intensity of physical activity can influence affective state, it was also assessed as a control variable. For this purpose, the Ratings of Perceived Exertion was used (Borg, 1982), which provides information about the subjective feeling of effort while doing physical activity. This can be rated on a 15-point scale ranging from 6 to 20. For example, the rating of 7 describes that an activity is perceived as “very, very light,” whereas 19 indicates the activity is felt to be “very, very hard.” For the current study, participants were asked to rate their overall (global) sense of exertion during the climbing session (“On the scale below, please describe your global sense of exertion during the climbing session so far”). In addition, we measured the local sensation of exertion in the forearms (“On the scale below, please describe your sense of exertion in your forearms during the climbing session so far”) based on the RPE. Psychometric parameters of the scale are given (Borg, 1982).

### 2.4 Climbing sessions

As this study followed a naturalistic approach, it was decided against matching the climbing conditions, e.g., in terms of route length or difficulty. Nevertheless, in order to ensure comparable conditions between climbing conditions, participants were given instructions at the end of the baseline measurement. It was emphasized that the focus was on sport climbing with rope and that outdoor climbing explicitly refers to sport climbing on rock. Subjects were instructed to warm up before each climbing session. The number of routes to be climbed and the duration of the long break were also predetermined. The grades of the routes were to be selected in such a way that the participants could climb them in one go, that is without rope strain or falling. In addition, different routes were always to be climbed in the lead. To control for social influences, participants were instructed to interact exclusively with their belay partners during the sessions. Furthermore, participants were instructed to perform the outdoor session only in dry conditions and at temperatures between 15 and 30 degrees Celsius.

The order in which interventions were conducted was relatively balanced: 42.5% of participants started with the indoor session first, and 57.5% began with the outdoor session (*n* = 40). In total, the indoor sessions (without warm-up) lasted 128.8 ± 56.8 min, and 5.4 ± 1.8 routes were climbed on average. Difficulties between 4b and 7c (median 6a) on the French scale were named as the highest grades that were climbed without any problems (“Indicate the highest grade of difficulty of the route that you climbed today without any problems [without rope load and fall]”). Subjects reported an average global perceived exertion of 13.8 ± 1.7, and local perceived exertion of 14.4 ± 2.3 for the indoor session.

The outdoor session (excluding warm-up) lasted an average of 176.4 ± 81.0 min, hereby a total of 4.9 ± 1.9 routes was climbed. The highest grades that were climbed without any problems were stated to be between 4a and 7b (median: 6a). Participants reported an average global perceived exertion of 13.4 ± 2.2, and local perceived exertion of 12.4 ± 2.6 for the outdoor session.

On average, there were 14.7 ± 46.8 days between the first and second climbing session (median & IQR = 6 days; *n* = 40).

### 2.5 Data processing and statistical analysis

SPSS (IBM Corp., US) 25th version was used for data processing. Two-factor repeated-measures ANOVAs served to analyze the research question of whether outdoor climbing has differential effects on affective state than indoor climbing. A 2 × 3 design with the within-subject factors environment (indoor - outdoor) and time (T1 - T2 - T3) was used. If the criterion of sphericity was not met as a basic requirement for the ANOVAs, the Greenhouse-Geisler correction was considered. Subsequently, simple contrast analyses were performed for the time factor with T1 as reference values. Dependent variables of the ANOVAs were the raw scores of the Feeling Scale and the Felt Arousal Scale. For the interpretation of the effects, the effect size partial *η*^2^ was used.

The research question of whether subjects had a stronger intention after the outdoor session than after the indoor session was tested using a dependent samples *t*-test after testing for normal distribution (Shapiro–Wilk-test). Cohen’s (1988) recommendations were considered to interpret the effect size *d*, according to which an effect of *d* = 0.20–0.49 can be classified as small, *d* = 0.50–0.79 as medium, and *d* ≥ 0.80 as strong.

The final research question, whether there is a positive relationship between expectancies and intention, was tested with multiple linear regressions. For this purpose, separate regression analyses were calculated depending on the environment. Predictors included in the model were self-efficacy and proximal outcome expectancies (at T1 and T3).

The significance level was set at *p* < 0.05, and two-sided tests for statistical significance were performed. Unless otherwise stated, all test values are presented as mean ± standard deviation.

## 3 Results

### 3.1 Sport climbing and affective state

**Figure 2:**
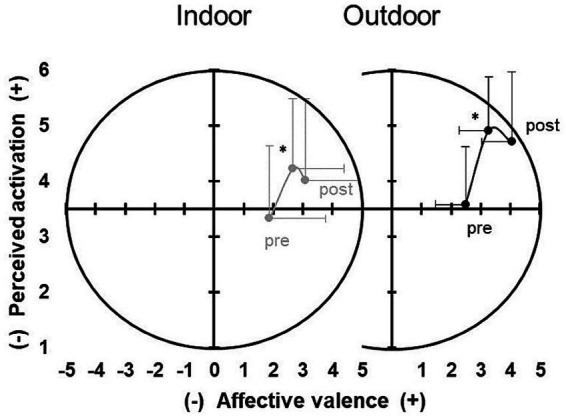
Changes in affective valence (horizontal axis) and perceived activation (vertical axis) over the climbing sessions visualized in the Circumplex Model of Affect. Error bars represent the standard deviation, * indicates significant main effects of the time factor for both affective valence and perceived activation.

The two-factor repeated-measures ANOVA for affective valence revealed significant main effects for the factors environment *F*(1, 47) = 9.08, *p* = 0.004, partial *η*^2^ = 0.16, and time *F*(1.73, 81.27) = 19.79, *p* < 0.001, partial *η*^2^ = 0.30. Contrast analyses indicated that in regard of the factor time, all test time points differed significantly from each other (*p* < 0.002). Scores in affective valence increased across all test time points during both the indoor and the outdoor session (Figure 2). Affective valence scores were always higher during the outdoor session. The interaction of the two factors environment and time was not significant *F*(2, 94) = 0.58, *p* = 0.563, partial *η*^2^ = 0.01.

For the ANOVA regarding perceived activation, a similar course of events was observed (Figure 2). Significant main effects were found for both the environment factor *F*(1, 47) = 10.89, *p* = 0.002, partial *η*^2^ = 0.19, and the time factor *F*(1.57, 73.61) = 27.82, *p* < 0.001, partial *η*^2^ = 0.37. Contrast analyses showed that regarding the factor time, the factor levels T1 and T3 as well as T1 and T2 differed significantly from each other (*p* < 0.001), but not T2 and T3 (*p* = 0.124). Perceived activation increased in the indoor session from T1 (3.3 ± 1.3) to T2 and decreased from T2 (4.2 ± 1.3) to T3 (4.0 ± 1.5) (Figure 2). During the outdoor session, a similar pattern emerged, with test scores again higher at each test time point than in the indoor session. The interaction of the factors environment and time was not significant for perceived activation *F*(2, 94) = 1.86, *p* = 0.162, partial *η*^2^ = 0.04.

### 3.2 Sport climbing and intention

A significant difference was found in mean scores of intention between the indoor (31.2 ± 4.7) and the outdoor (32.5 ± 4.1) condition, *t*(47) = −2.21, *p* = 0.032. Participants reported significantly stronger intention after the outdoor session than after the indoor session, *d* = −0.32; 95% CI [−0.61, −0.03]. This effect can be categorized as small.

### 3.3 Relationship between expectancies and intention

Regarding the regression model for the indoor session, *F*(3, 44) = 15.89, *p* < 0.001, proximal outcome expectancies, measured both before, *p* = 0.010, *β* = 0.33, 95% CI [0.08, 0.58], and after the climbing session, *p* < 0.001, *β* = 0.51, 95% CI [0.26, 0.76], were significantly associated with behavioral intention (Table 2). The results suggest a positive relationship, i.e., that an increase in expectancies was associated with an increase in intention. The association between intention and self-efficacy was not significant, *p* = 0.449, *β* = −0.09, 95% CI [−0.31, 0.14]. *R^2^* was 0.52 for the indoor model.

**Table 2:** Multiple linear regression with the predictors self-efficacy expectancy and proximal outcome expectancies (at T1 & T3) on behavioral intention as a criterion variable for the indoor condition.

Variable	M	SD	B	SE (B)	*β*	95% CI (*β*)		*p*
						LL	UL	
Self-efficacy expectancy	4.9	0.8	−0.50	0.65	−0.09	−0.31	0.14	0.449
Proximal outcome expectancies (T1)	30.8	4.9	0.31	0.12	0.33	0.08	0.58	0.010
Proximal outcome expectancies (T3)	31.4	4.3	0.55	0.13	0.51	0.26	0.76	< 0.001

T1, measurement time point before the climbing session; T3, measurement time point after the climbing session; M, mean, SD, standard deviation; B, regression coefficient B; SE (B), standard error of regression coefficient; *β,* standardized beta weight; 95% CI, 95% confidence interval of *β*; LL, lower limit, UL, upper limit; *p*, significance value. *R*^2^ is 0.52; *n* = 48.

In the model for the outdoor session, none of the variables were significantly associated with behavioral intention, *F*(3, 44) = 0.73, *p* = 0.541. *R*^2^ of the model was 0.05 (Table 3).

**Table 3:** Multiple linear regression with the predictors self-efficacy expectancy and proximal outcome expectancies (at T1 & T3) on behavioral intention as a criterion variable for the outdoor condition.

Variable	M	SD	B	SE (B)	*β*	95% CI (*β*)		*p*
						LL	UL	
Self-efficacy expectancy	4.9	0.8	−0.25	0.76	−0.05	−0.35	0.25	0.741
Proximal outcome expectancies (T1)	32.3	3.9	0.02	0.18	0.02	−0.32	0.36	0.912
Proximal outcome expectancies (T3)	33.3	3.6	0.23	0.19	0.21	−0.13	0.55	0.223

T1, measurement time point before the climbing session; T3, measurement time point after the climbing session; M, mean, SD, standard deviation; B, regression coefficient B; SE (B), standard error of regression coefficient; *β*, standardized beta weight; 95% CI, 95% confidence interval of *β*; LL, lower limit; UL, upper limit; *p*, significance value. *R*^2^ is 0.05; *n* = 48.

## 4 Discussion

### 4.1 Main findings

The aim of the present study was to compare outdoor and indoor sport climbing in terms of affective state, expectancy, and intention to better understand the role of the natural environment regarding physically active behavior. In summary, the results indicate that outdoor climbing seems to be generally associated with a more positive affective state than indoor climbing. However, stronger changes in the scores over the course of the climbing sessions did not appear between outdoor and indoor sport climbing. A stronger intention for future climbing after the outdoor session was reported by the participants. Furthermore, it appears that especially in indoor climbing, proximal outcome expectancies are significantly related to post-session intention for future climbing.

### 4.2 Sport climbing and affective state

The data of the present study show that sport climbing had significant positive effects on affective state with an increase of affective valence over the climbing sessions. With respect to the Circumplex Model, test scores for affective state of both conditions (indoor - outdoor) are largely located in the upper right quadrant, which Ekkekakis et al. (2011) suggest makes future maintenance of a behavior more likely. Climbing-specific literature has already reported that climbing appears to have positive effects on affective state. For example, Frühauf et al. (2020) found that a single therapeutic climbing session can lead to an increase in affective valence as well as perceived activation in children and adolescents with mental health disorders. Also, Bichler et al. (2022) illustrated that the exercise programs climbing and nordic walking had more beneficial effects on affective state (including short-term anxiety-relieving effects) in individuals with anxiety disorder and PTSD, compared to social contact sessions without exercise. Thus, the results of the present study are basically consistent with such findings. However, it must be added that comparable studies often take place in a therapeutic setting. Moreover, they usually focus on therapeutic climbing, which cannot be equated with recreational sport climbing. Studies examining climbing as a recreational sport in non-clinical samples are scarce in this context, which makes comparison with the literature somewhat difficult.

That green exercise is associated with a more positive affective state compared to indoor exercise is largely consistent with previous research (Thompson Coon et al., 2011; Gladwell et al., 2013). One possible explanation for why physical activity in natural environments has a more favorable effect on affective state compared to urban environments is provided by the attention restoration theory (ART). According to ART, such effects are associated with natural stimuli, so-called “soft fascinations” (e.g., moving trees, clouds in the sky). These “soft fascinations” bind the attention without effort and provide the feeling of “being away.” This allows for a restoration of attentional capacities, which is also associated with an improvement in affective state (Kaplan and Kaplan, 1989). An important element to answer the research question was an interaction of the factors environment and time, which was identified by some authors using other modes of physical activity. For example, Niedermeier et al. (2017) reported that mountain hiking resulted in greater benefits in terms of affective state (valence and activation) than indoor walking on a treadmill or in comparison to a sedentary control condition. For both, clinical and non-clinical context, there are hardly any studies that considered environmental effects in climbing in terms of affective state, which is why it is difficult to make a comparison with the literature. However, the present data did not provide any evidence for such an interaction. Thus, outdoor sport climbing appears to be associated with better affective state overall, but it does not seem to have a stronger effect on affective state than indoor climbing. This finding must be put in the context that in outdoor climbing, the distance between bolts usually is longer compared to the distance between protections in the gym. This might result in an increased stress or anxiety response outdoors, potentially lowering the affective valence outdoors. However, we found an overall higher affective valence outdoors compared to indoors. We speculate that affective response outdoors might be even higher, when the distance between fixed protections is standardized between both conditions.

The fact that the participants reported a more positive affective state even before the start of the outdoor climbing session (compared to the indoor session) could be explained as follows. Questionnaire T1 was to be completed immediately before the session, so it must be assumed that most participants were already in the gym or at the crag. In order to get to an outdoor climbing area, an approach is generally necessary which already requires physical activity in a natural environment. This in turn could be the reason for the higher values regarding affective state (outdoor session) already before the start of the session. In this context, the first few minutes of green exercise seem to have a large influence on affective state, which might explain the effects for shorter approaches as well (Barton and Pretty, 2010). Of course, it is conceivable that physical activity was also performed prior to the indoor session, e.g., when arriving by bicycle. However, the state of research indicates stronger effects associated with physical activity that is performed in natural environments (Niedermeier et al., 2017). Basically, the effects of physical activity on psychological variables seem to be modulated by the characteristics of the environment. Gidlow et al. (2016) showed that an outdoor walk in both urban and natural environments had positive effects on participants’ mood. However, restorative experiences and greater cognitive benefits were found to be more pronounced after walks in more natural environments. It is even possible that the mere sight of natural surroundings contributed to cause the offset in affective state scores at T1 between the conditions (Yeo et al., 2020). It is also conceivable that the higher values in affective state before the outdoor climbing session result from anticipatory processes regarding nature or natural scenery (Haluza et al., 2014).

The present study contributes to the existing literature by showing partly similar environmental effects in sport climbing. However, the technical aspect of climbing should also be considered, as climbing requires a high degree of concentration or focus and other cognitive processes (Leichtfried, 2015). It is possible that the characteristics of climbing facilitate a distractive effect on, for example, negative thoughts or everyday brooding (Morgan, 1985).

### 4.3 Sport climbing and intention

The finding that individuals report greater intention after performing physical activity in natural environments, has been described before. In this context, Focht (2009) found that even a 10-minute walk can lead to improvements in affective state, both indoors and outdoors. Participants reported in connection with a walk outdoors significantly higher affective state, enjoyment, and stronger intention for future walks than in connection with an indoor walk. Focht (2009) additionally showed that the variable enjoyment correlated significantly with intention for future walking both indoors and outdoors. He also found significant correlations between affective state (among others Feeling Scale) and enjoyment. This allows the consideration that the higher values in the intention may be due to the connection with enjoyment. This could be a possible explanation for the findings of the present study. Thompson Coon et al. (2011) also concluded, based on their systematic review, that physical activity performed in natural environments seems to be associated with greater enjoyment and a stronger intention to repeat the activity in the future. Still, research evidence is inconclusive about whether outdoor activities are associated with higher intention (Rogerson et al., 2016; Lahart et al., 2019). This may partly be explained by the fact that existing reviews often included studies with methodological flaws, which in turn relativizes the significance of their findings (Thompson Coon et al., 2011; Lahart et al., 2019). For this reason, further research is needed. Nevertheless, the results of the present study support a stronger intention to repeat the activity after green exercise.

### 4.4 Relationship between expectancies and intention

Data analysis showed significant, positive associations between proximal outcome expectancies and intention in the context of indoor climbing.

That expectancies reflecting proximal affective outcomes are particularly useful for predicting intention or physical activity has already been described (Rhodes and Conner, 2010; Gellert et al., 2012). In this context, Klusmann et al. (2016) argue that proximal outcome expectancies (affective outcomes) are by nature less susceptible to interference and therefore more likely to occur than distal ones, which is why proximal expectancies are particularly important for the maintenance of a behavior or behavior change. Social outcomes, on the other hand, depend on the behavior of others, and whether physical outcomes occur is usually only apparent over time. Incidentally, in this context, the authors identified the degree to which expectancies were met as an important predictor. In the regression model for the outdoor session, however, no such relationship between proximal outcome expectancies and intention was observed. The low degree of explained variance in the latter model suggests that there may be other variables that are more predictive of intention (outdoors) but were not included in this model. Since a person’s motivation to perform a particular behavior is influenced by both person- and situation-related factors, it may also be necessary to consider the motives for which people generally go climbing. Such motives as power, performance, or social connection (Wegner, 2020) should be collected in combination with the incentives (experience of nature, independence from weather) offered by the environment. It can be considered that these differ between individuals.

Both from a theoretical perspective and confirmed in scientific research, the variable self-efficacy is assumed to be a good predictor for physical activity or intention (Gao et al., 2007; Barg et al., 2012; Zhang et al., 2019). The results of the present study do not provide support for such a relationship. One explanatory approach may be methodological. The HAPA by Schwarzer (1992, 2004) was developed to predict or explain health-promoting and -harming behaviors. This model states that a certain degree of self-efficacy must always be present for a behavior to occur and considers different forms of self-efficacy (Schwarzer, 2004); however, these have a phase-specific effect. For the implementation and maintenance of a behavior, maintenance and recovery self-efficacy are important (volitional phase), but for intention formation, action self-efficacy is particularly relevant (motivational phase). Action self-efficacy stands for the belief in one’s own ability to perform a certain behavior. Maintenance self-efficacy reflects a person’s belief in being able to deal with obstacles that may cause the intended action to fail. Similarly, recovery self-efficacy reflects expectancies of overcoming setbacks and recovering from failed attempts to realize the target behavior (Zhang et al., 2019). That action self-efficacy is associated with intention has been confirmed in previous research (Barg et al., 2012; Zhang et al., 2019). The self-efficacy scale used in the present study was chosen because it was designed for the sport context. However, this does aim at barriers or obstacles that may interfere with the target behavior, which suggests that this was measuring maintenance self-efficacy rather than action self-efficacy. This in turn might explain why no relationship with intention was found. Possibly, the scale used to measure self-efficacy is also less suitable for sport climbing in general due to some items. For example, the item “I am sure that I will still be able to perform a planned sports activity (sport climbing) even if the weather is bad” may have proved problematic. It can be assumed that it is less likely that one will go outdoor climbing in bad weather, because this is very unpleasant and even dangerous. For subsequent research, a more suitable item selection that is equally well suited for indoor and outdoor sport is recommended.

## 5 Strengths and limitations

To the best of our knowledge, the present research is the first study to analyze the concepts of expectancy, intention, and affective state in the context of sport climbing considering the environment.

Basically, there are some limitations that should be considered when interpreting the results. The data showed that the instructions were not always strictly followed and that there were in some cases deviations (e.g., number of routes climbed). It must therefore be assumed that certain confounding variables also had a varying effect on the participants. In addition, it is possible that certain environmental factors, such as temperature, varied across climbing sessions and could therefore have had different effects on the parameters, such as affective state. With such a naturalistic design, it is generally important to note that, compared to a strictly controlled design, the control of potential confounding variables can only be considered to a limited extent. As this was an online survey, it cannot be guaranteed that the information provided by the participants was always truthful. Furthermore, the study focused only on positive expectancies. Klusmann et al. (2016) suggest that negative expectancies also have an effect on health behaviors, but of a more negative nature. Moreover, the scale used to measure self-efficacy seems to be less suitable for climbing. Especially with regard to intention formation, the use of a scale that explicitly measures action self-efficacy would probably have been more purposeful here. Finally, it must be pointed out that the sample studied was made up of young, predominantly male, German-speaking adults with a relatively high level of education, which limits generalization to other samples.

Besides these limitations, however, this study is characterized by several strengths. One of them concerns the chosen study design. By using a within-subject design with multiple test time points, it was possible to reduce inter-individual variability and to control for potential daily fluctuations in affective state. Another advantage is that most data were collected directly during the climbing session; memory problems or biases were thus minimized. In addition, the data were collected online under individual responsibility, which meant that the normal climbing procedure was hardly disturbed. This favors that the data reflect an authentic situation close to everyday life (no experimenter effect).

## 6 Conclusion

The study showed that the participants’ affective state improved during both an indoor and an outdoor climbing session. Even though this effect was comparable between the conditions, outdoor climbing seems to be generally associated with a more positive affective state and a stronger intention for future climbing. However, it must be added that indoor climbing also went along with an increase in affective state and relatively strong intention. It is assumed that a higher affective state as well as a stronger intention regarding physical activity make it more likely that the corresponding behavior will be performed also in the future. All in all, the results at least partly suggest environmental effects on behaviorally relevant variables during climbing. Outdoor climbing might provide more favorable characteristics for physically active behavior compared to indoor climbing. Building on this, health psychologists or public health professionals who focus on increasing their clients’ physical activity levels may encourage their clients to choose outdoor climbing over indoor climbing.

Due to the chosen naturalistic design, some confounding variables could only be controlled to a limited extent; given these methodological limitations, these results should not be generalized without restriction (c.f. Lahart et al., 2019). Further research is needed in this area in order to be able to make more reliable statements. Future research should integrate a more controlled design with experimenters present. In this context, it seems appropriate to match the different climbing conditions, for example in terms of route length, slope or degree of difficulty. In addition, further potentially important variables could be collected, such as the preferred environment and motivational variables. In the context of outcome expectancies, it may be useful to also determine an individual weighting of the outcome (value), as postulated by the classical expectancy × value models. It would also be interesting to compare expectancies and actual experiences, i.e., the extent to which expectancies are actually fulfilled.

Finally, the following must be added: Although affective state and intention seem to be related to whether future physically active behavior will be performed, this does not necessarily mean that high scores automatically lead to more physically active behavior. Future studies should include follow-up analyses to investigate the extent to which the respective constructs actually predict physically active behavior at a later point in time.

## Data availability statement

The raw data supporting the conclusions of this article will be made available by the authors, without undue reservation.

## Ethics statement

The studies involving humans were approved by the Review Board of the Institute of Sport Science of the University of Innsbruck. The studies were conducted in accordance with the local legislation and institutional requirements. The participants provided their written informed consent to participate in this study.

## Author contributions

BH: Conceptualization, Data curation, Formal analysis, Investigation, Methodology, Writing – original draft. MN: Conceptualization, Writing – review & editing. MK: Conceptualization, Methodology, Supervision, Writing – review & editing.
